# Clinical standards for diagnosis, treatment and prevention of post-COVID-19 lung disease

**DOI:** 10.5588/ijtld.23.0248

**Published:** 2023-10-01

**Authors:** D. Visca, R. Centis, E. Pontali, E. Zampogna, A.-M. Russell, G. B. Migliori, C. Andrejak, M. Aro, H. Bayram, K. Berkani, J. Bruchfeld, J. M. Chakaya, J. Chorostowska-Wynimko, B. Crestani, M. P. Dalcolmo, L. D’Ambrosio, A-T. Dinh-Xuan, S. Duong-Quy, C. Fernandes, J.-M. García-García, A de Melo Kawassaki, L. Carrozzi, M. A. Martinez-Garcia, P. Carreiro Martins, M. Mirsaeidi, Y. Mohammad, R. N. Naidoo, N. Neuparth, L. Sese, D. R. Silva, I. Solovic, T. M. Sooronbaev, A. Spanevello, N. Sverzellati, L. Tanno, S. Tiberi, T. Vasankari, E. Vasarmidi, M. Vitacca, I. Annesi-Maesano

**Affiliations:** 1 Division of Pulmonary Rehabilitation, Istituti Clinici Scientifici (ICS) Maugeri, Istituto di Ricovero e Cura a Carattere Scientifico (IRCCS), Tradate; 2 Department of Medicine and Surgery, Respiratory Diseases, University of Insubria, Varese; 3 Respiratory Diseases Clinical Epidemiology Unit, Istituti Clinici Scientifici Maugeri, IRCCS, Tradate; 4 Department of Infectious Diseases, Galliera Hospital, Genoa, Italy; 5 Faculty of Health and Life Sciences, University of Exeter, Exeter; 6 Royal Devon University Hospitals NHS Trust, Exeter; 7 North Bristol NHS Trust, Bristol, UK; 8 Respiratory Department, Centre Hospitalier Universitaire Amiens Picardie, Amiens; 9 Unité de Recherche 4294, Agents Infectieux, Résistance et Chimiothérapie, Picardie Jules Verne University, Amiens; 10 GREPI (Group pour la Recherche et enseignement en pneumo-infectiologie) Work group of French society of respiratory diseases, Paris, France; 11 Finnish Lung Health Association (FILHA), Helsinki, Finland; 12 Department of Pulmonary Medicine, Koc University Research Center for Translational Medicine, Koc University School of Medicine, Istanbul, Turkey; 13 Pierre de Soleil Clinic, Respiratory Rehabilitation, Vetraz Monthoux, France; 14 Department of Infectious Diseases, Karolinska University Hospital, Stockholm; 15 Division of Infectious Diseases, Department of Medicine, Karolinska Institutet, Stockholm, Sweden; 16 Department of Medicine, Therapeutics and Dermatology, Kenyatta University, Nairobi, Kenya; 17 Department of Clinical Sciences, Liverpool School of Tropical Medicine, Liverpool, UK; 18 Department of Genetics and Clinical Immunology, National Institute of Tuberculosis and Lung Diseases, Warsaw, Poland; 19 Université Paris Cité, Physiopathologie et épidémiologie des maladies respiratoires, Institut national de la santé et de la recherche médicale (INSERM), Paris; 20 Assistance Publique des Hôpitaux de Paris (APHP), Hôpital Bichat, Service de Pneumologie A, FHU APOLLO, Paris, France; 21 Hélio Fraga Reference Center, Fundação Oswaldo Cruz (Fiocruz), Rio de Janeiro, RJ, Brazil; 22 Public Health Consulting Group, Lugano, Switzerland; 23 Service de Physiologie-Explorations Fonctionnelles, APHP, Hôpital Cochin, Université Paris Cité, Paris, France; 24 Respiratory Department, Lam Dong Medical College, Dalat, Vietnam; 25 Heart Institute, Cardio-pulmonology Department, University of Sao Paulo, Sao Paulo, SP, Brazil; 26 Tuberculosis Research Programme (PII-TB), Sociedad Española de Neumología y Cirugía Torácica, Barcelona, Spain; 27 Serviço de Pneumologia, Instituto do Câncer do Estado de São Paulo (ICESP) e do ambulatório de Doenças Pulmonares Intersticiais, Hospital das Clínicas, Universidade de São Paulo, São Paulo, SP, Brazil; 28 Department of Surgical, Medical, and Molecular Pathology and Critical Care Medicine, University of Pisa, Pisa; 29 Pulmonary Unit, Cardiothoracic and Vascular Department, University Hospital of Pisa, Pisa, Italy; 30 Respiratory Department, University and Polytechnic La Fe Hospital, Valencia; 31 Centro de Investigación Biomédica en Red, Respiratory Disorders, Madrid, Spain; 32 Allergy and Clinical Immunology Department, Dona Estefânia Hospital, Centro Hospitalar Universitário de Lisboa Central, Lisbon; 33 NOVA Medical School-Comprehensive Health Research Center, Lisbon, Portugal; 34 Division of Pulmonary and Critical Care, University of Florida, Jacksonville, FL, USA; 35 Al Sham private University, Faculty of Medicine and Pharmacy, Damascus and Latakia; 36 Centre for Research on Chronic Respiratory Diseases, Tishreen University, Lattakia, Syria; 37 Discipline of Occupational and Environmental Health, University of KwaZulu-Natal, Durban, South Africa; 38 Department of Physiology and Functional Explorations, Hôpital Avicenne, INSERM, Unité mixte de recherche 1272 Hypoxia and the Lung, Université Sorbonne Paris Nord, Bobigny; 39 Department of Pneumology, Centre Constitutif de référence des maladies pulmonaires rares, Hôpital Avicenne, Bobigny, France; 40 Faculdade de Medicina, Universidade Federal do Rio Grande do Sul, Porto Alegre, RS, Brasil; 41 National Institute for TB, Lund Diseases and Thoracic Surgery, Vysne Hagy, Catholic University, Ruzomberok, Slovakia; 42 Department of Respiratory Medicine, National Center for Cardiology and Internal Medicine, Bishkek, Kyrgyzstan; 43 Department of Medicine and Surgery, University of Parma, Parma, Italy; 44 Institut Desbrest of Epidemiology and Santé Publique, INSERM & Montpellier University, Montpellier and Department of Allergic and Respiratory Diseases, Montpellier University Hospital, Montpellier, France; 45 Blizard Institute, Barts and the London School of Medicine and Dentistry, Queen Mary University of London, London, UK; 46 FILHA, Helsinki; 47 University of Turku, Department of Pulmonary Diseases and Clinical Allergology, Turku, Finland; 48 Department of Respiratory Medicine and Laboratory of Molecular and Cellular Pneumonology, School of Medicine, University of Crete, Heraklion, Greece; 49 ICS Maugeri IRCCS, Respiratory Rehabilitation of the Institute of Lumezzane, Brescia, Italy

**Keywords:** long COVID, SARS-COV-2, post-COVID conditions, pulmonary rehabilitation, quality of life

## Abstract

**BACKGROUND:**

The aim of these clinical standards is to provide guidance on ‘best practice’ care for the diagnosis, treatment and prevention of post-COVID-19 lung disease.

**METHODS:**

A panel of international experts representing scientific societies, associations and groups active in post-COVID-19 lung disease was identified; 45 completed a Delphi process. A 5-point Likert scale indicated level of agreement with the draft standards. The final version was approved by consensus (with 100% agreement).

**RESULTS:**

Four clinical standards were agreed for patients with a previous history of COVID-19: Standard 1, Patients with sequelae not explained by an alternative diagnosis should be evaluated for possible post-COVID-19 lung disease; Standard 2, Patients with lung function impairment, reduced exercise tolerance, reduced quality of life (QoL) or other relevant signs or ongoing symptoms ≥4 weeks after the onset of first symptoms should be evaluated for treatment and pulmonary rehabilitation (PR); Standard 3, The PR programme should be based on feasibility, effectiveness and cost-effectiveness criteria, organised according to local health services and tailored to an individual patient’s needs; and Standard 4, Each patient undergoing and completing PR should be evaluated to determine its effectiveness and have access to a counselling/health education session.

**CONCLUSION:**

This is the first consensus-based set of clinical standards for the diagnosis, treatment and prevention of post-COVID-19 lung disease. Our aim is to improve patient care and QoL by guiding clinicians, programme managers and public health officers in planning and implementing a PR programme to manage post-COVID-19 lung disease.

## Abstract (fr)

**CONTEXTE:**

L’objectif de ces normes cliniques est de fournir des conseils sur les « meilleures pratiques » en matière de diagnostic, de traitement et de prévention des maladies pulmonaires post-COVID-19.

**MÉTHODES:**

Un groupe d’experts internationaux représentant des sociétés scientifiques, des associations et des groupes actifs dans le domaine des maladies pulmonaires post-COVID-19 a été constitué ; 45 d’entre eux ont participé à un processus Delphi. Une échelle de Likert en 5 points a permis d’indiquer le niveau d’accord avec les projets de normes. La version finale a été approuvée par consensus (100% d’accord).

**RÉSULTATS:**

Quatre normes cliniques ont été approuvées pour les patients ayant des antécédents de COVID-19 : Norme 1, les patients présentant des séquelles non expliquées par un autre diagnostic doivent être évalués en vue d’une éventuelle maladie pulmonaire post-COVID-19 ; Norme 2, les patients présentant une altération de la fonction pulmonaire, une diminution de la tolérance à l’effort, une réduction de la qualité de vie (QoL) ou d’autres signes pertinents ou des symptômes persistants, quatre semaines ou plus après l’apparition des premiers symptômes, doivent être évalués en vue d’un traitement et d’une réadaptation pulmonaire (PR, de l’anglais ‘*pulmonaire rehabilitation*’) ; Norme 3, le programme de PR doit être basé sur des critères de faisabilité, d’efficacité et de rentabilité, organisé en fonction des services de santé locaux et adapté aux besoins individuels des patients ; et Norme 4, chaque patient qui suit et termine un programme de PR doit être évalué pour déterminer son efficacité et avoir accès à une session de conseil/éducation à la santé.

**CONCLUSION:**

Il s’agit du premier ensemble consensuel de normes cliniques pour le diagnostic, le traitement et la prévention des maladies pulmonaires post-COVID-19. Notre objectif est d’améliorer les soins et la qualité de vie des patients en guidant les cliniciens, les responsables de programmes et les responsables de la santé publique dans la planification et la mise en œuvre d’un programme de relations publiques pour la prise en charge des maladies pulmonaires post-COVID-19.

Early in the COVID-19 pandemic, it became apparent that many surviving patients continued to suffer the consequences of acute disease even after being considered ‘cured’.^1–4^ It appears that COVID-19 has an impact on previous chronic respiratory disorders, and previous respiratory disorders can also impact the course of COVID-19 disease and any subsequent sequalae.^5–7^ In 2021, the WHO defined post-COVID-19 sequelae as the presence of symptoms (fatigue, shortness of breath, cognitive dysfunction) in a patient with a history of probable or confirmed SARS-CoV-2 infection, usually 3 months from the onset of COVID-19, with symptoms lasting more than 2 months and not explained by an alternative diagnosis.^8,9^ The National Institute for Health and Care Excellence (NICE) guidelines describe ‘long COVID’ as signs and symptoms that continue or develop after acute COVID-19, including ongoing symptomatic COVID-19 (4–12 weeks) and post-COVID-19 syndrome (≥12 weeks).^10^ A recent meta-analysis supported female sex as a risk factor for long COVID,^11,12^ which is also associated with previous medical comorbidities, such as cardiovascular diseases, hypertension, pulmonary disease, diabetes, obesity and organ transplantation.^13^ The US Centers for Disease Prevention and Control (CDC) uses the umbrella term ‘post-COVID conditions’ to include a wide range of health consequences present for ≥4 weeks after infection with SARS-CoV-2.^8,14^ Having survived the acute phase of COVID-19, millions of people around the world experienced prolonged symptoms for several months.^15–18^ These symptoms were identified in 10–35% of people managed in an outpatient setting during the initial disease, and in up to 80% of people hospitalised with more severe initial illness.^19,20^ The most frequent symptoms include fatigue, anosmia, dysgeusia, headache, attention disorder, hair loss, anxiety, tachycardia, palpitations, brain fog, dizziness, cough, confusion and dyspnoea/dysfunctional breathing pattern.^12,19–22^

Here, we discuss how these health consequences can be addressed by implementing clinical standards for the diagnosis, treatment and prevention of post-COVID-19 lung disease. These standards build on a statement by the European Respiratory Society (ERS) describing follow-up strategies for long COVID (which acknowledged that the evidence for follow-up care was limited at the time of publication in 2023).^23^ Individual patients with COVID-19 may experience different degrees of sequelae: mild, moderate or severe. Generally, sequelae that include persistent but reversible symptoms with no need for treatment are classified as mild. Sequelae that need action in terms of further investigation and treatment, even if usually treatable and reversible, are defined as moderate. Severe sequelae include deep vein thrombosis, strokes or pulmonary embolism.^4,24,25^ Furthermore, rare severe sequelae are those presenting with chronic organ failure, such as cardiovascular events, including myocarditis, postural orthostatic tachycardia syndrome, renal failure or pulmonary fibrosis. Pathophysiological processes impacting long COVID include organ damage, resulting from acute phase infection, complications from a dysregulated inflammatory state and inadequate antibody response against SARS-CoV-2.^26^ Although it is acknowledged that long COVID affects several organs and/or body systems, here we primarily focus on lung damage. In summary, the term post-COVID-19 lung disease as used here includes signs and symptoms from sequelae, which continue or develop after the acute phase, and feature all the relevant conditions described above.^8–10,14^

## AIM OF THE CLINICAL STANDARDS

Our aim is to provide expert guidance on ‘best practice’ for diagnosis, treatment and prevention of post-COVID-19 lung disease. Developing standards for a new, incompletely characterised disease is challenging, as we have only limited long- to medium-term experience of the condition. However, the type of lung damage and their symptoms are similar to the sequelae or complications observed in other respiratory diseases, including post-TB lung disease (PTLD).^27–31^ We have therefore extrapolated from data on other respiratory conditions and previously published clinical standards to inform our approach.^32–35^ Evidence on post-COVID lung disease is still lacking in areas such as benefits/risks evaluations, and costs and cost-analysis. We present an ‘optimal’ set of standards (recommendations for the best possible approach), but acknowledge that implementing this approach may be difficult in some settings. We therefore include adaptations for special settings and situations to indicate how the approach can be modified as needed.

This consensus-based document describes the following process for patients with a previous history of COVID-19:

1. Patients with sequelae not explained by an alternative diagnosis should be evaluated for possible post-COVID-19 lung disease (Standard 1).
2. Patients with lung function impairment, reduced exercise tolerance, reduced quality of life (or other relevant signs or ongoing symptoms) ≥4 weeks after the onset of the first symptoms should be evaluated for treatment and pulmonary rehabilitation (PR) (Standard 2).
3. The PR programme should be based on feasibility, effectiveness and cost-effectiveness criteria, organised according to local health services and tailored to the patient’s needs (Standard 3).
4. Each patient undergoing and completing PR should be evaluated to determine its effectiveness and have access to a counselling/health education session (Standard 4).

In addition, consensus-based research priorities were identified.

## METHODS

A panel of 63 global experts were invited to represent the main scientific societies, associations and groups active in post-COVID-19 lung disease. Of the 63 experts, 4 declined, and 12 did not respond after one invitation reminder. All respondents (*n* = 47) were asked to comment on an initial set of six draft standards developed by a core team (*n* = 7) of researchers via a Delphi process. Of these, 45 researchers provided valid answers; following the Delphi process, the six draft standards were reduced to four. The final writing panel included the following 40 experts: COVID/infectious diseases and respiratory clinicians (*n* = 32), public health specialists, including respiratory epidemiologists (*n* = 4), a physiotherapist, an occupational physician, a paediatrician and a methodologist. A 5-point Likert scale (5: high agreement; 1: low agreement) was used to indicate agreement with the standards. At the first Delphi round, agreement was high, with a median value of >4 (for all standards). Based on substantial initial agreement, the expert panel developed an initial draft, which underwent five rounds of revisions. The final version was approved by consensus (100% agreement). As evidence in this field is rapidly accumulating, this document will be updated once significant new evidence emerges.

## STANDARD 1

**Patients with sequelae not explained by an alternative diagnosis should be evaluated for possible post-COVID-19 lung disease.**

A wide range of symptoms and clinical outcomes occur in people with varying degrees of illness from post-COVID-19 conditions. Evaluation should include assessment of respiratory conditions for persistent or new respiratory symptoms, measurements of exercise capacity, functional status or health-related quality of life (QoL)^23^ and other conditions (asthenia, insomnia, depression) (Table 1).^36–39^ In special settings and situations, this evaluation can be simplified and/or modified to include a set of core examinations with the aim to identify patients with sequelae at risk for deterioration and those likely to benefit most from PR.

**Table 1:** Selected assessment tools for evaluating people with post-COVID-19 at the end of the acute phase

Condition	Essential and conditional examinations/investigations	Adaptation to special setting and situations
Respiratory signs and symptoms^36,37^	Oxygen saturation Modified Medical Research Council Dyspnoea Scale Spirometry (full pulmonary lung function tests where feasible) SaO_2_ monitoring on activity	Oxygen saturation Modified Medical Research Council Dyspnoea Scale Spirometry (FEV_1_ and FVC) or peak expiratory flow meter if spirometry not available
Exercise capacity (at least one of the exercises)	6-minute walk test In alternative: 10 m walk test Sit-to-stand test 2-min step test	6-minute walk test In alternative: 10 m walk test Sit-to-stand test 2-min step test
Functional status or quality of life (at least one)^38^	Patient-centred outcomes measures for COVID-19 Post-Covid-19 Functional Status Scale EuroQol-5D Short Form 36	Patient-reported outcomes measurement information system (e.g., cognitive function 4a) Post-Covid-19 Functional Status Scale EuroQol-5D Short Form 36
Mental well-being (at least one)	Generalised Anxiety Disorder-7 Patient Health Questionnaire-9 Hospital Anxiety and Depression Scale Post Traumatic Stress Disorder Symptom Scale Screen for Posttraumatic Stress Symptoms PTSD Checklist for DSM-5 Impact of Event Scale-Revised	Generalised Anxiety Disorder-7 Patient Health Questionnaire-9 Hospital Anxiety and Depression Scale Post Traumatic Stress Disorder Symptom Scale
Other symptoms/conditions^39^	Chalder Fatigue Scale Fatigue Severity Scale Insomnia Severity Index (ISI) Connective Tissue Disease Screening Questionnaire	Chalder Fatigue Scale

FEV_1_ = forced expiratory volume in 1 sec; FVC = forced vital capacity; EuroQOL = Euro Quality of Life; SaO_2 _= oxygen saturation; PTSD = post-traumatic stress disorder; DSM = Diagnostic and Statistical Manual of Mental Disorders.

The relationship between thoracic images and persisting abnormalities in lung function requires further study. In patients with normal chest radiography and oxygen saturation, computed tomography (CT, or high-resolution CT, HRCT) imaging of the chest might have some role for assessing pulmonary disease.^40,41^ Thoracic imaging of pulmonary sequelae of COVID-19 from 10 to 12 months after the acute event has revealed fibrotic changes, including parenchymal bands, irregular interfaces and reticular opacities, with or without honeycomb-like changes.^41–43^ Where available, CT/HRCT can be used to identify post-COVID pulmonary abnormalities,^40,41,43,44^ considering that the type of abnormality may vary according to the severity of the acute phase, duration of hospitalisation and need for mechanical ventilation.^40,41,45–47^ For patients who may require imaging based on clinical findings, symptom management and a rehabilitation plan can often be initiated simultaneously with the imaging workup. However, it is important to point out that CT imaging is not available in many countries, and be aware that patients could present with clinical symptoms and radiological signs evoking post-COVID-19 without a previous diagnosis of COVID-19.

Patients, especially those with severe COVID-19, may develop micro-clots that persist months after the initial infection.^48^ Assessment and treatment with anti-platelet therapies and or anticoagulants are outside the scope of this document and is fully addressed in guidelines for antithrombotic treatment for COVID‐19.^49^ Antibody tests, when available, could help to confirm diagnosis, especially when reverse transcription polymerase chain reaction or rapid antigen test was not done for the initial diagnosis of COVID-19, although many individuals will since have been vaccinated.^23^

A comprehensive assessment should be done in individuals experiencing new or ongoing symptoms at least 4–12 weeks or more after the acute phase (see Table 1) to identify subjects suitable for a PR programme^10,50,51^ involving nurses, physiotherapists and psychologists. The presence of respiratory signs and symptoms will aid the identification of patients with respiratory sequelae at risk of deterioration and most likely to benefit from PR as discussed below.

## STANDARD 2

**Patients with lung function impairment, reduced exercise tolerance, reduced QoL or other relevant signs or ongoing symptoms ≥4 weeks after the onset of the first symptoms, should be evaluated for treatment and PR.**

PR is described as a ‘comprehensive intervention based on a thorough patient assessment, followed by patient-tailored therapies that include, but are not limited to, exercise training, education and behaviour change, designed to improve the physical and psychological condition of people with chronic respiratory disease and to promote the long-term adherence to health-enhancing behaviours’.^52^ Treatment of post-COVID-19 lung disease should focus on managing the patient’s symptoms, improving function and health-related QoL. Recent evidence supports the need for prompt referral to rehabilitation in patients hospitalised for COVID-19 disease, while for non-hospitalised patients, referral should follow an assessment by healthcare workers or a watch and wait/self-management approach for at least 6 weeks. An overall evaluation of functional impact and physical performance in post-COVID-19 patients should focus on symptoms limiting daily activities, pulmonary function tests (PFT), diffusion capacity of the lung for carbon monoxide (DLCO), blood gas analysis (BGA), pulse oximetry at rest and on exertion, QoL, anxiety and depression (Table 2).^9,17,23,36,37,52–77^ Patients presenting with lung function impairment (airflow obstruction or restriction or mixed and/or impaired DLCO and/or gas exchange), reduced exercise tolerance and related impairment in QoL and other relevant signs or symptoms (fatigue, exhaustion, asthenia or weakness; respiratory symptoms such as dyspnoea, cough, chest pain; respiratory failure at rest and/or on exertion) should be evaluated for PR.

**Table 2:** Indications for pulmonary rehabilitation

Indications	Essential and conditional examinations/investigations	Adaptation to special setting and situations
Reported symptoms: fatigue (or exhaustion or asthenia or weakness) and or dyspnoea, cough, chest pain^17,52,54,58,59,70^	Modified Borg Scale (0–10) or Visual Analogue Scale In alternative or addition: Fatigue Severity Scale Chalder Fatigue Scale Checklist Individual Strength Medical Research Council Barthel Dyspnoea Leicester Cough Questionnaire	Modified Borg Scale (0–10) or Visual Analogue Scale
Impaired health-related quality of life^57,59,62^	EuroQol five dimensions, and St George’s Respiratory Questionnaire	EuroQol five dimensions, and/or St George’s Respiratory Questionnaire
Impaired ADL/functional status^52,54,57,59,61,62^	Barthel’s Index, and COPD Assessment Test In alternative or addition: Medical Research Council muscle Post-COVID-19 Functional Status DePaul Post-Exertional Malaise Questionnaire Functional impairment Checklist Functional independence measure Fried frailty index	Barthel’s Index, and/or COPD Assessment Test
Impaired exercise capacity^52,54,57–59,62,68,71–74^	Six-min walking test and/or Sit to stand test In alternative or addition: Cardiopulmonary exercise test One repetition maximum Short Physical Performance Battery 2-min step test 10 m walk test Maximal voluntary contraction Handgrip strength	Six-min walking test and/or Sit to stand test 2-min step test
Presence of comorbid conditions, including COPD, asthma, bronchiectasis, pulmonary fibrosis, pulmonary hypertension, cardiac impairment, autonomic nervous system disfunction^52,57^	Clinical history, evaluation for additional tests/examinations	Clinical history, evaluation for additional tests/examinations
Impaired pulmonary function^23,36,37,57,58,73–76^	Spirometry and Lung volume if available Diffusion capacity for carbon monoxide	Spirometry or PEF if not available
Impaired blood gas analysis and/or exercise-induced desaturation^52–54,57,59,69^	Blood gas analysis, and Pulse oxymetry	Pulse oxymetry
Reported anxiety and depression^77^	Generalized Anxiety Disorder-7 and/or Patient Health Questionnaire-9 In alternative or addition: Hospital Anxiety and Depression Scale	Generalized Anxiety Disorder-7, and/or Patient Health Questionnaire-9

COPD = chronic obstructive pulmonary disease; PEF = peak expiratory flow; ADL = activity of daily life.

A pilot study from 2021 evaluated COVID-19 patients 6 months after discharge from hospital and showed that persistent dyspnoea was associated with reduced physical fitness.^68^ Patients in need of early and effective PR are those who had suffered from the severe acute forms of COVID-19 and those with persistent abnormal chest X-ray or CT or reduced DLCO.^17^

Patients with COVID-19 admitted to intensive care unit (ICU) may develop a post-intensive care syndrome (PICS), with impaired physical, exercise-induced oxygen desaturation, muscle weakness and reduced mobility.^53^ Such patients are likely to benefit from PR. Finally, patients experiencing severe lung impairment during COVID-19 acute illness, prone to altered PFT and 6-min walk test (6MWT) results, are a subgroup of patients that could also benefit from PR.^69^

## STANDARD 3

**The PR programme should be based on feasibility, effectiveness and cost-effectiveness criteria, organised according to local health services and tailored to the patient’s needs.**

PR is a multidisciplinary, non-pharmacological intervention aiming to improve symptoms, health status, and exercise capacity and reduce disability in post-acute and chronic respiratory diseases.^52^ There is strong evidence that PR is also successful when multiple comorbidities are present.^78^ PR should be completed within the framework of a multidisciplinary programme. Based on the experience with other respiratory conditions, this programme consists of physical and exercise therapy, along with psychology, nursing and medical interventions, as needed. Symptoms reported by post-COVID-19 patients include breathlessness, anxiety, depression, fatigue, reduced lung and muscle capacity and comorbidities, highlighting the emerging role of PR as an effective intervention in post-COVID-19 lung disease.^57,62,79–86^ A dysfunctional or abnormal breathing pattern has also been reported by patients with long COVID, both at rest and during exercise testing. In patients with post-COVID-19 dysautonomia, such as postural orthostatic tachycardia syndrome, breathing exercises in an upright position can be challenging and a supine position during exercise should instead be recommended.^87–89^

PR programmes for long COVID have been proposed in different settings according to the local organisation of health services, ranging from in- and out-patients, home and tele-rehabilitation.^57,58,62,73,74,77,90^ Such programmes should be tailored to the patient’s needs and include some minimum and essential requirements.^91^ These include areas of rehabilitation and interventions focused on the baseline assessment, exercise training, education to support self-management, psychological counselling as required, and recommendations for home-based exercise and physical activity,^50,57,58,62,90–92^ with a programme length ranging from 1–12 weeks.^73,74,77^ A follow-up plan is recommended to maintain benefits from PR and evaluate additional clinical problems arising at a later stage. PR programmes cannot be planned without considering the local organisation of health services, such as hospital settings, community, one-stop-shop clinics, out-patient clinics, or integrated into primary care.^93–100^ They should also be organised according to feasibility, effectiveness and cost-effectiveness criteria,^91,101^ and adapted to the context and resources available in different settings to ensure that they are as accessible as possible for patients, including children and adolescents.^91,102,103^ PR programmes can also be delivered in person, through tele-rehabilitation, depending on each patient’s individual context.

Both during and after the COVID-19 pandemic, there has been an accelerated interest in alternative forms of exercise training, such as yoga and dance, integrated with or as an adjunct to PR programmes. Dance improves motor function (balance, strength, exercise capacity and QoL) in older patients in other disease contexts and is being evaluated as an adjunct to PR in respiratory conditions.^104^ Yoga, incorporating breathing exercises, stretching and gentle chair and standing exercises, is offered by patient advocacy organisations such as the Irish Lung Fibrosis Association (https://ilfa.ie/blog/online-yoga-class/) as an adjunct to PR and warrants further evaluation. The benefits of structured singing programmes for lung health, which are offered outside the traditional health service model, are also emerging.^105^

Our understanding of the benefits of PR is mostly derived from the experience of people affected by chronic respiratory diseases, especially chronic obstructive pulmonary disease (COPD), where it has proven to be cost-effective in preventing hospital readmission.^106^ PR could accelerate recovery in post-COVID-19 patients, but further studies are needed to identify effective and cost-effective strategies to deliver PR in the different settings. The core components of a PR programme are summarised in Table 3.^16,57,58,62,73,74,77,82,90,107–113^

**Table 3:** Summary of the core components of a rehabilitation programme

Components and indication	Core interventions	Adoptions to special setting and situations
Aerobic exercise: endurance training^57,58,62,90^ Impaired exercise capacity, limited by dyspnoea, fatigue and or other symptoms. Restriction in daily life activities	Treadmill and/or cycle-ergometer 30 min 2–5 times/week for 4–8 weeks Continuous or interval training Low-intensity (40–60%) or high-intensity (60–80%) set according to: maximal heart rate (220-age), or maximal oxygen consumption, or maximal watt, or the 6-min walking test, or symptom limited (target Borg scale ≤4) In or out-patients or tele-monitoring Suggest maintenance programme	Free walks 30 min 2–5 times/week for 4–8 weeks Intensity set according to perceived symptoms (target Borg scale ≤4) Out-patients or home setting Suggest maintenance programme
Strength training: upper and lower extremities^57,62,73,107,108^ Sarcopenia, reduced strength of peripheral muscles. Lower muscle weakness with risk for falls. Impaired activities of daily living involving the upper extremities (including dressing, bathing and household tasks)	Free weights (dumbbells and ankle-brace) 20–30 min 2–5 times/week for 4–8 weeks, 2–3 set of 6–12 repetitions Intensity set to 80% of maximal voluntary contraction or one maximum repetition and/or adjusted on perceived muscles fatigue (target Borg scale ≤4) In or out-patients or tele-monitoring Suggest maintenance programme	Free weights (dumbbells and ankle-brace) 20–30 min 2–5 times/week for 4–8 weeks, 2–3 set of 6–12 repetitions Intensity set according to perceived muscles fatigue (target Borg scale ≤4) Out-patients or home setting Suggest maintenance programme
Education^57^ Impaired/reduced self-efficacy and collaborative self-management	Structured and comprehensive educational programmes Age-specific, gender-sensitive, delivered in the local language and extended to family and/or caregivers Individual or group sessions: 15–60 min Importance of physical activity and exercise to improve quality of life Maintaining results achieved with PR (follow-up plan) Advantages/importance of smoking cessation and risk of comorbidities (e.g., diabetes, etc.) Importance of adhering to medical prescriptions in terms of management of comorbidities and vaccinations Achieving an optimal healthy life style	Structured and comprehensive educational programmes Age specific, gender-sensitive, delivered in the local language and extended to family and/or care-givers Individual or group sessions 15–60 min Importance of physical activity and exercise to improve quality of life Maintaining results achieved with PR (follow-up plan) Advantages/importance of smoking cessation and risk of comorbidities (e.g., diabetes, etc.) Importance of adhering to medical prescriptions in terms of management of comorbidities and vaccinations Achieving an optimal healthy life style
Components and indication	Additional interventions	Adoptions to special setting and situations
Breathing exercise^57,62,74,109^ Dynamic hyperinflation Resting tachypnoea Dyspnoea	Adaptive breathing strategies Yoga breathing Singing for lung health Pursed-lips breathing Computer-aided breathing feedback: 2–4 times per week for 30 min each	Adaptive breathing strategies Yoga breathing Singing for lung health Pursed-lips breathing: 2–4 times per week for 30 min each
Airway clearance techniques^57,109,110^ Difficult to remove secretions or mucous plugs. Frequent bronchial exacerbations (≥2/year) Concomitant diagnosis of bronchiectasis	Choose the technique suitable for the subject among those available, based on respiratory capacity, mucus rheology, collaboration and patient preferences: 15–30 min one or more times/day Choose the duration of treatment based on chronic (long-term) or acute problem (short-term) Suggest maintenance programme when needed	Choose the technique suitable for the subject among those available, based on respiratory capacity, mucus rheology, collaboration and patient preferences: 15–30 min one or more times/day Choose the duration of treatment based on chronic (long-term) or acute problem (short-term) Suggest maintenance programme when needed
Inspiratory Muscle training^107,108^ Impaired respiratory muscle function, altered respiratory mechanics, decreased chest wall compliance or pulmonary hyperinflation	Load threshold devices, seated and using a nose clip: Intensity/load set at 30–60% of maximal inspiratory pressure Interval training, 3 sets with10 breaths, followed by 1-min break between each set. 15–20 min 2–5 times/week for 4–8 weeks	Not applicable
Psychological support^16,57,77,82^ Depression, anxiety and cognitive dysfunction	Psychological assessment Psychological support Relaxation technique Consider self-help group	Psychological assessment Psychological support Relaxation technique Consider self-help group
Nutritional support^111–113^ Body composition abnormalities	Nutritional assessment Tailored treatment from foods and medical supplements	Nutritional assessment Tailored treatment from foods and medical supplements Need for financial incentives and transportation access should be evaluated

PR = pulmonary rehabilitation.

## STANDARD 4

**Each patient undergoing and completing pulmonary rehabilitation should be evaluated to determine its effectiveness and have access to a counselling/health education session.**

Studies with strong evidence on the efficacy of interventions, in particular, PR and long-term monitoring for post-COVID-19 lung disease are lacking. No guidelines for post-COVID-19 interventions exist, and standardised methods for evaluating their efficacy have not been developed. Standard 4 includes a short description on how to evaluate the effectiveness of PR, comparing core variables pre- and post-rehabilitation. The standard also suggests how to organise the patient’s follow-up to maintain or improve the results achieved, organised according to feasibility and cost-effectiveness criteria, based on the local organisation of health services and tailored to an individual patient’s needs. For patients with long COVID, the objective of PR is to increase functional independence. This can be evaluated (as for all respiratory diseases) by a significant improvement in PFT results^50,114,115^ and respiratory symptoms. In this context, the strongest independent predictors of persistent respiratory impairment with need for follow-up^114,115^ are patients with COVID-19 presenting with dyspnoea 3 weeks after hospital discharge and those with impaired gas exchange on admission to hospital. Data show that functional capacity and QoL should not be neglected,^114,116,117^ and a multidisciplinary rehabilitation strategy is essential to address this.

Community-based PR interventions and monitoring may also be beneficial during pandemics.^114^ Community health workers can contribute to the COVID-19 response by providing screening, facilitating referrals, arranging support for home care, staffing community-based isolation centres, and being involved in surveillance, contact tracing, service delivery to people with disabilities, home visits, outreach activities and advocacy campaigns.^23,114^

## PREVENTION

There are points in the sequence of events spanning SARS-COV-2 infection to reporting prolonged and persistent suffering due to post-COVID sequelae, at which there is the possibility of a preventive intervention/strategy. Primary prevention is based on avoiding SARS-COV-2 infection; the basic interventions that can be implemented are well acknowledged: face-masks, hand sanitisation, personal distancing, appropriate ventilation of indoor settings and vaccination.^118–123^ The secondary area of prevention includes intervening during acute disease to prevent sequelae: pharmacological treatment with antivirals and corticosteroids, selective prophylactic anticoagulants, critical care and early rehabilitation in acute care.^9,49,118,124^ Tertiary prevention interventions, including disability limitation and rehabilitation, are post-acute disease. Quaternary interventions include activities that help in preventing treatment and vaccine side effects.

## PRIORITIES FOR FUTURE RESEARCH

Examples of future research priorities^125^ are summarised below.

### Diagnosis

- Identification of predictors of post-COVID lung disease: patient characteristics (disabilities, comorbidities, genetics), biomarkers, extrinsic factors (biological, including air pollution; psychological and social), radiological patterns during acute disease, manifestations and severity of COVID disease;
- Development and evaluation of diagnostic tools and algorithms for post-COVID lung disease;
- Evaluation of the effectiveness of multidisciplinary, post-COVID-19 clinics vs. traditional GPs’ assessment with referral to organ specialists;
- Definition of criteria for severity of post-COVID disorders;
- Assessment of knowledge on post-COVID lung disease of health workers in primary care centres and public hospital clinics;
- Development and validation of tools for auditing activities on post-COVID lung disease;
- Evaluation of long-term consequences of post-COVID lung disease, with special focus on bronchiectasis and pulmonary fibrosis;
- Impact of post-COVID in developing countries.

### Treatment

- Exploration of role (efficacy, safety, dosing, etc.) of the different drugs for the various manifestations of post-COVID lung disease: antibiotics, corticosteroids, anti-fibrotic drugs (e.g., pirfenidone or nintedanib), anti-histamines, combination therapy of anti-platelet therapies and anti-coagulants in the later variants of SARS‐CoV‐2;
- Evaluation of the long-term impact of PR on disability and QoL;
- Assessment of rehabilitation knowledge and needs, aimed at elaborating training programmes;
- Exploration of optimal treatments and their outcomes in patients with immunodeficiencies;
- Exploration of role (efficacy, safety, etc.) of non-conventional, non-pharmacological interventions (singing, yoga and movement, and dance);
- Efficacy of anti-COVID-19 vaccines as treatment of post-COVID lung disease;
- Identification of cost-effective strategies to deliver PR;
- Establishing standards for assessing fitness to work and return to normal duties.

### Prevention

- Efficacy of anti-COVID-19 vaccines on the prevention of post-COVID lung disease;
- Efficacy of other preventive measures on the prevention of post-COVID lung disease: daily facial masks, hand washing/disinfection, limitations on re-occurrence of disease;
- Identification of factors that can be potential targets for interventions to prevent post-COVID disease;
- Evaluation of the impact of early and personalised PR in the acute phase of COVID-19 to prevent post-COVID sequelae;
- Exploring strategies and developing guidelines to prevent venous thromboembolism (VTE) occurrence and post-COVID VTE recurrence;
- Evaluation of the role of antifibrotic therapy for the prevention of pulmonary fibrosis in patients with reduced DLCO 12 weeks after SARS-CoV-2 infection;
- Strategies and approaches for the prevention of exacerbations and bronchiectasis;
- Identification of pharmaceutical, physical and psychological interventions in those with infection to prevent progress to post-COVID-19 disease;
- Telemedicine for video-observed PR;
- Inclusion of educational modules on the effect of air pollution and the benefits of smoking cessation.

## CONCLUSION

A significant number of people continue to require care after recovering from acute COVID-19.^4,21,24,91^ There is preliminary, but increasing evidence that PR interventions are useful for post-COVID-19 lung disease; however, implementation of these programmes remains slow. Beyond adopting what is already acknowledged as effective, there is a strong need to accumulate and evaluate the increasing evidence on the use of PR for post-COVID-19 lung disease and investigate innovative PR interventions. There is also a need for research on the underlying mechanisms of post-COVID-19 pulmonary sequelae. As the currently available evidence is only modest, we need periodical evaluations of PR to guide evidence-based implementation of adequate measures to assess and manage post-COVID-19 lung disease.

Within the ‘optimal’ approach to the management of post-COVID-19 lung disease these Clinical Standards promote, digital services may help to reduce costs and the time lost to travel in some settings. Digital services supporting both treatment, management and PR of patients include asynchronous clinical communications, real-time virtual care, messaging, telephony or video conferencing, virtual health assessments and medication review.^23,126^
